# Supplementary material for: Genome editing in primary cells and in vivo using viral-derived Nanoblades loaded with Cas9-sgRNA ribonucleoproteins
Source: Nat Commun. 2019 Jan 3;10:45. doi: 10.1038/s41467-018-07845-z (PMC6318322; doi:10.1038/s41467-018-07845-z)

Supplementary Information

Genome editing in primary cells and in vivo using viral-derived “Nanoblades” loaded with Cas9/sgRNA ribonucleoproteins

Mangeot et al.

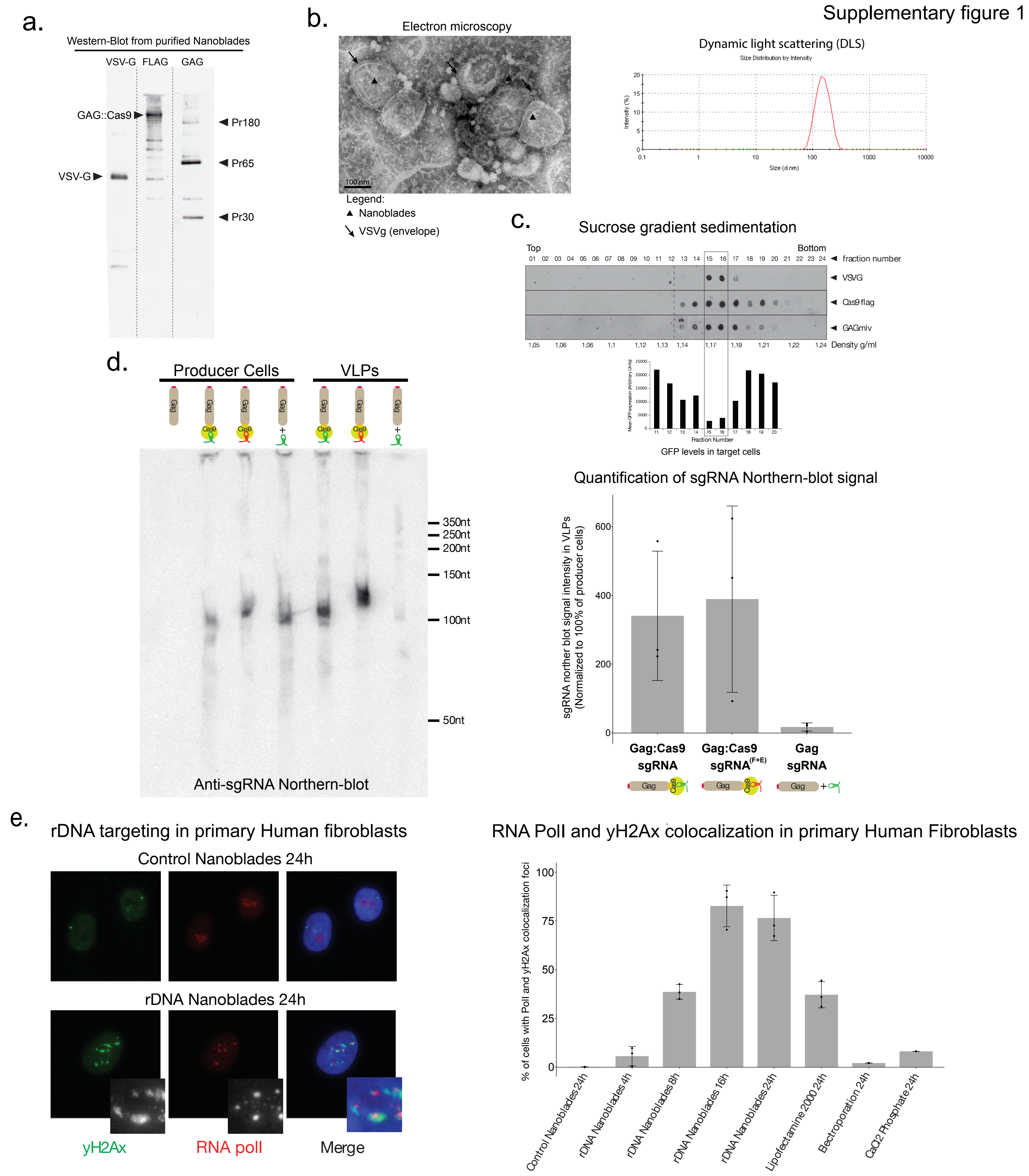

Supplementary figure 1. Molecular, structural and biochemical characterization of Nanoblades. a. Western blot analysis of proteins from purified Nanoblades using anti VSV-G, anti-Flag and anti-Gag antibodies. **b.** Electron microscopy and dynamic light scattering analysis of purified Nanoblades. **c.** Sucrose sedimentation analysis of Nanoblades targeting the GFP coding sequence. Each fraction of the sucrose gradient was analysed by Western-blotting to monitor the presence of VSV-G, Cas9 and Gag. Bottom panel, fractions 11 to 20 were collected and incubated with immortalized mouse macrophages stably expressing GFP. GFP expression was then measured by FACS 96h after transduction. **d.** Left panel, Northern-blot analysis of total RNA extracted from producer cells and purified Nanoblades using a radioactive probe complementary to the conserved region of the sgRNA. From left to right, producers cells expressing Gag only or Gag::Cas9 + sgRNA or Gag::Cas9 + optimised sgRNA or Gag + sgRNA and VLPs obtained from cells expressing Gag::Cas9 + sgRNA or Gag::Cas9 + optimised sgRNA or Gag + sgRNA. Right-panel, quantification of the Northern-blot signal (n=3, error bars correspond to standard deviation). **e.** Left panel, immunofluorescence analysis of γ-H2AX (green), RNA pol I (red) in primary human fibroblasts 24 hours after being transduced with control Nanoblades or with Nanoblades targeting ribosomal DNA genes. Right panel, quantification of γ-H2AX and RNA Pol I colocalization foci in primary fibroblasts at different times after Nanoblades transduction or after classical DNA transfection methods (n=3 for all conditions but electroporation and CaCl2 transfection which were done in n=1, error bars correspond to standard deviation).

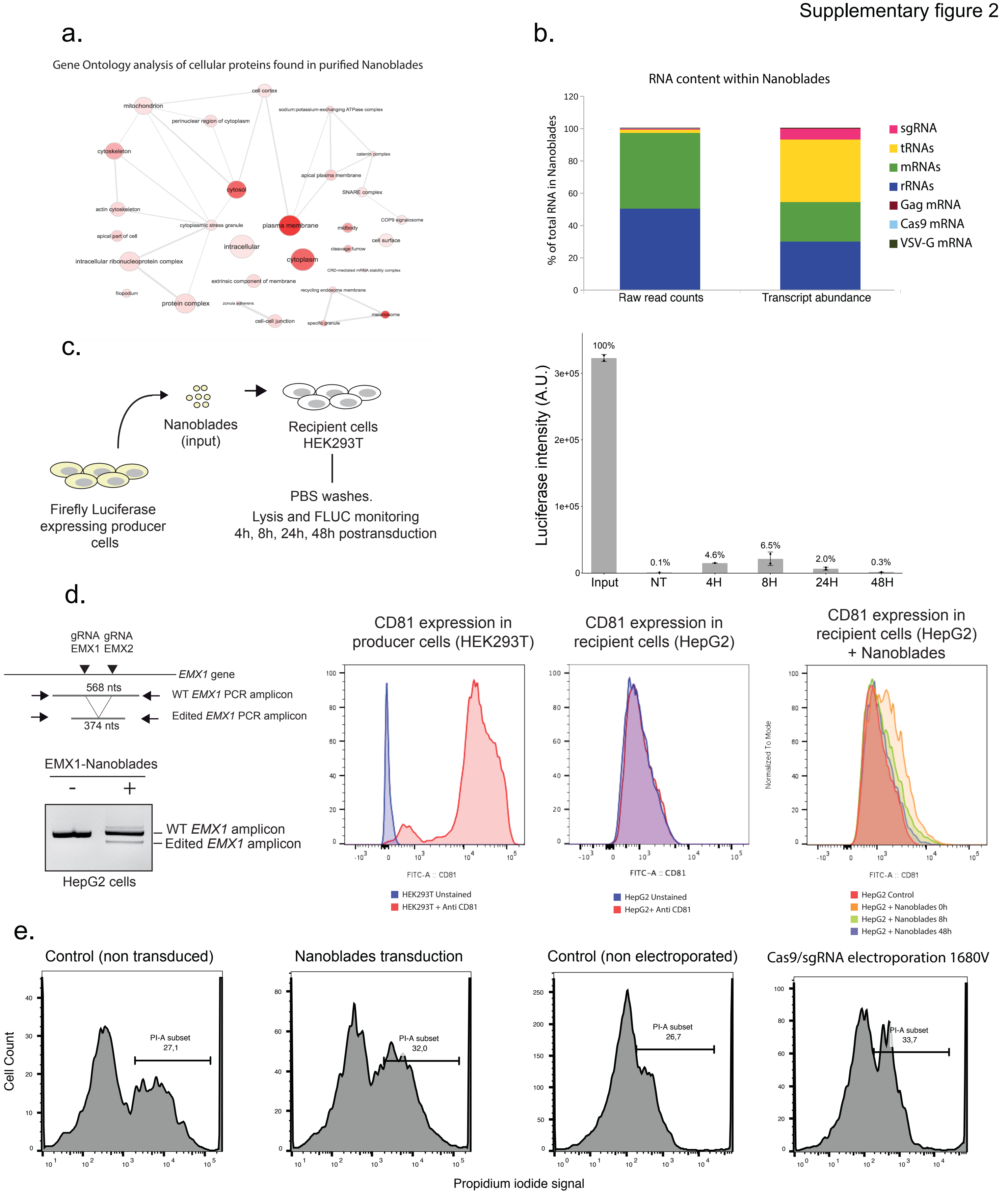

Supplementary figure 2. Protein and RNA content of purified Nanoblades. a. Gene ontology analysis of proteins identified by Mass Spectrometry in Nanoblades. **b.** Relative quantification of all RNAs found within Nanoblades by high-throughput sequencing. **c.** Firefly luciferase transfer from producer to recipient cells (HEK293T cells). Left panel, scheme of the procedure. Right panel, luciferase activity levels in input Nanoblades and in recipient cells at 4, 8, 24 and 48 hours after transduction. **d.** Top panel, flow cytometry analysis of CD81 expression in producer HEK293T cells (left panel), recipient HepG2 cells (middle panel) and recipient cells at different times after transduction with Nanoblades (right panel). Bottom panel, editing at the EMX1 locus in HepG2 tested by PCR amplification using primers flanking the two target sites. **e.** Flow cytometry analysis of primary mouse bone marrow cells incubated with propidium iodide after Nanoblades transduction or electroporation of the Cas9/sgRNA RNP. Displayed numbers correspond to the percentage of cells positive for propidium iodide staining.

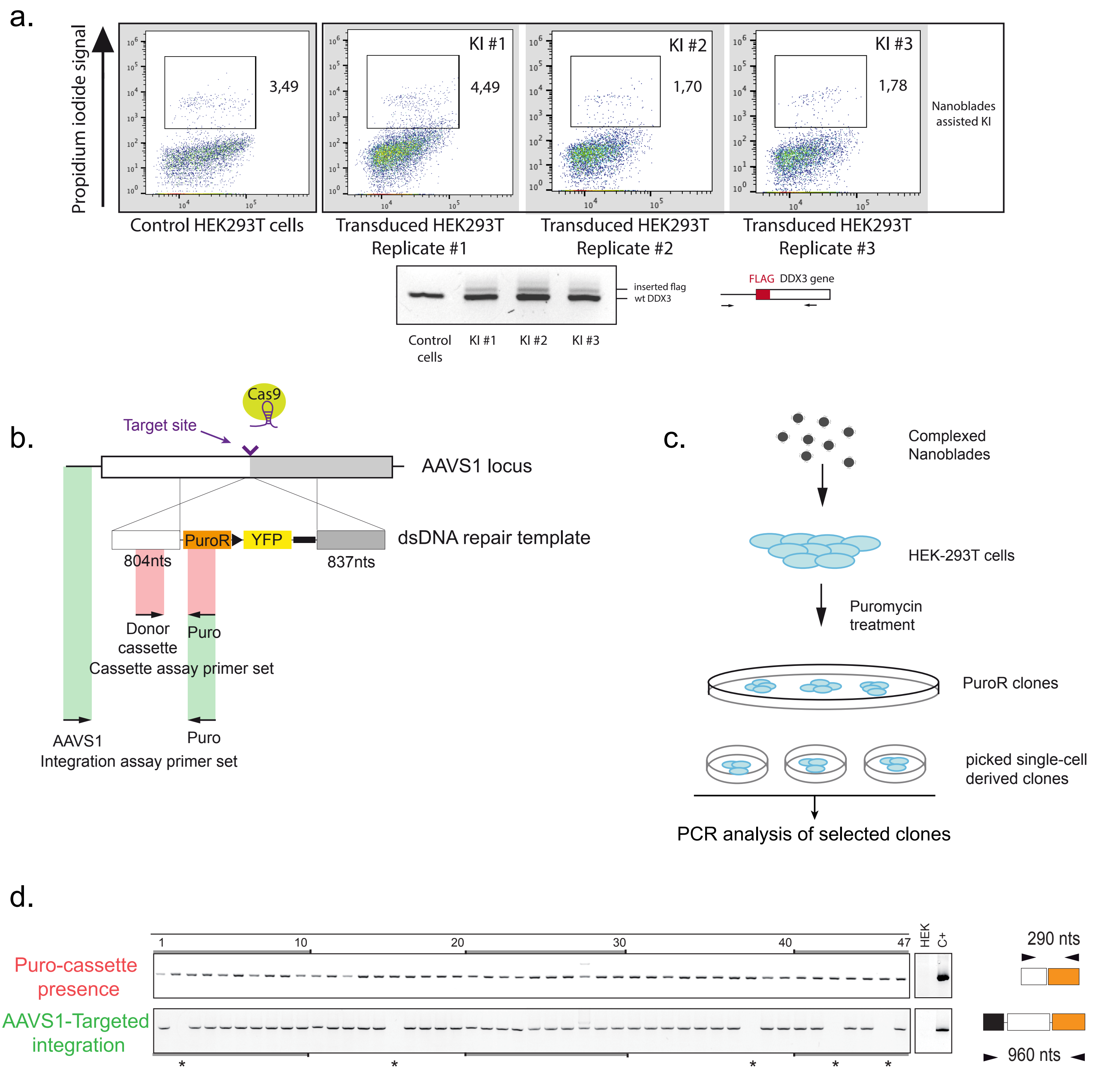

Supplementary figure 3. "All-in-one" Knock-in of a puromycin cassette in the AAVS1 locus. a. Top panels, flow cytometry analysis of HEK293T cells incubated with propidium iodide after transduction with Nanoblades targeting the DDX3 locus complexed with a ssDNA donor oligonucleotide bearing the Flag-DDX3 sequence. Displayed numbers correspond to the percentage of cells positive for propidium iodide staining. Bottom panel, FLAG-tag insertion at the DDX3 locus assessed by PCR using primers flanking the Flag sequence (Insertion PCR assay) described in Figure3a. **b.** Scheme of the knock-in strategy and the dsDNA puromycin cassette. **c.** Scheme of the transduction and clonal selection strategy. Briefly, 1.105 HEK293T cells were transduced with "all-in-one" Nanoblades targeting the AAVS1 locus and complexed with the dsDNA puromycin resistance cassette. After transduction, cells were incubated with puromycin until individual resistant clones were visible. **d.** 47 resistant clones were obtained in total that were isolated to generate monoclonal cell lines. Both, presence and targeted insertion of the puromycin cassette in the AAVS1 locus, were assayed by PCR (using respectively, the Donor Cassette and Puro oligomers, or the AAVS1 and Puro oligomers depicted in "b.". PCR analysis of the Puromycin cassette and the AAVS1 targeted insertion in the 47 puromycin resistant clones and in non-transduced HEK293T cells (HEK) and in a positive control for which targeted insertion was assayed by Sanger sequencing (C+).

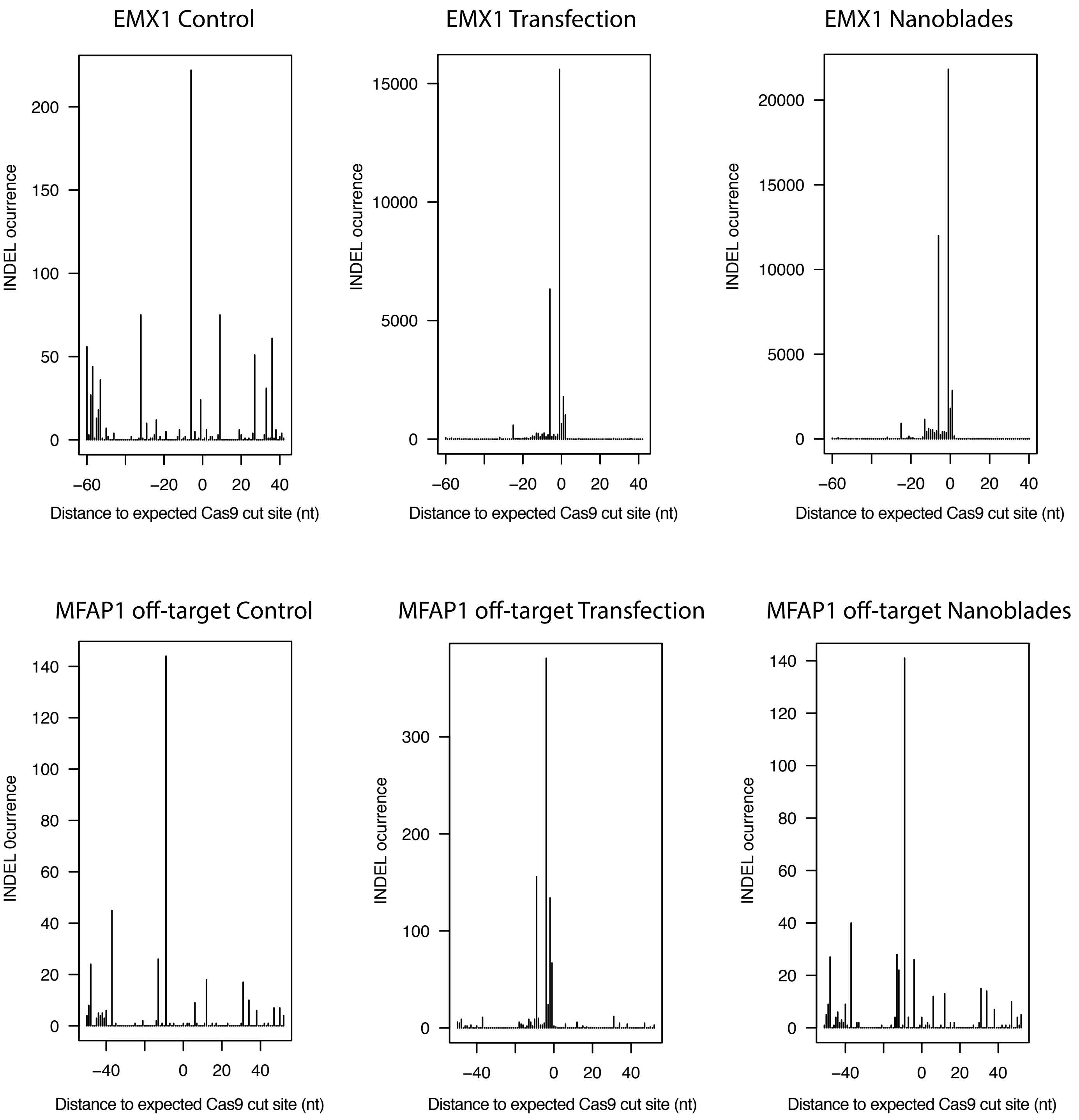

Supplementary figure 4. Position of INDELs detected at the EMX1 on-target site and the MFAP1 off-target site. The frequency of INDELs detected in high-throughput sequencing reads were plotted for each position of the EMX1 (Top panels) and MFAP1 (Bottom panels) loci using the expected Cas9 cut site (3nt upstream the PAM site) as the 0 offset position.

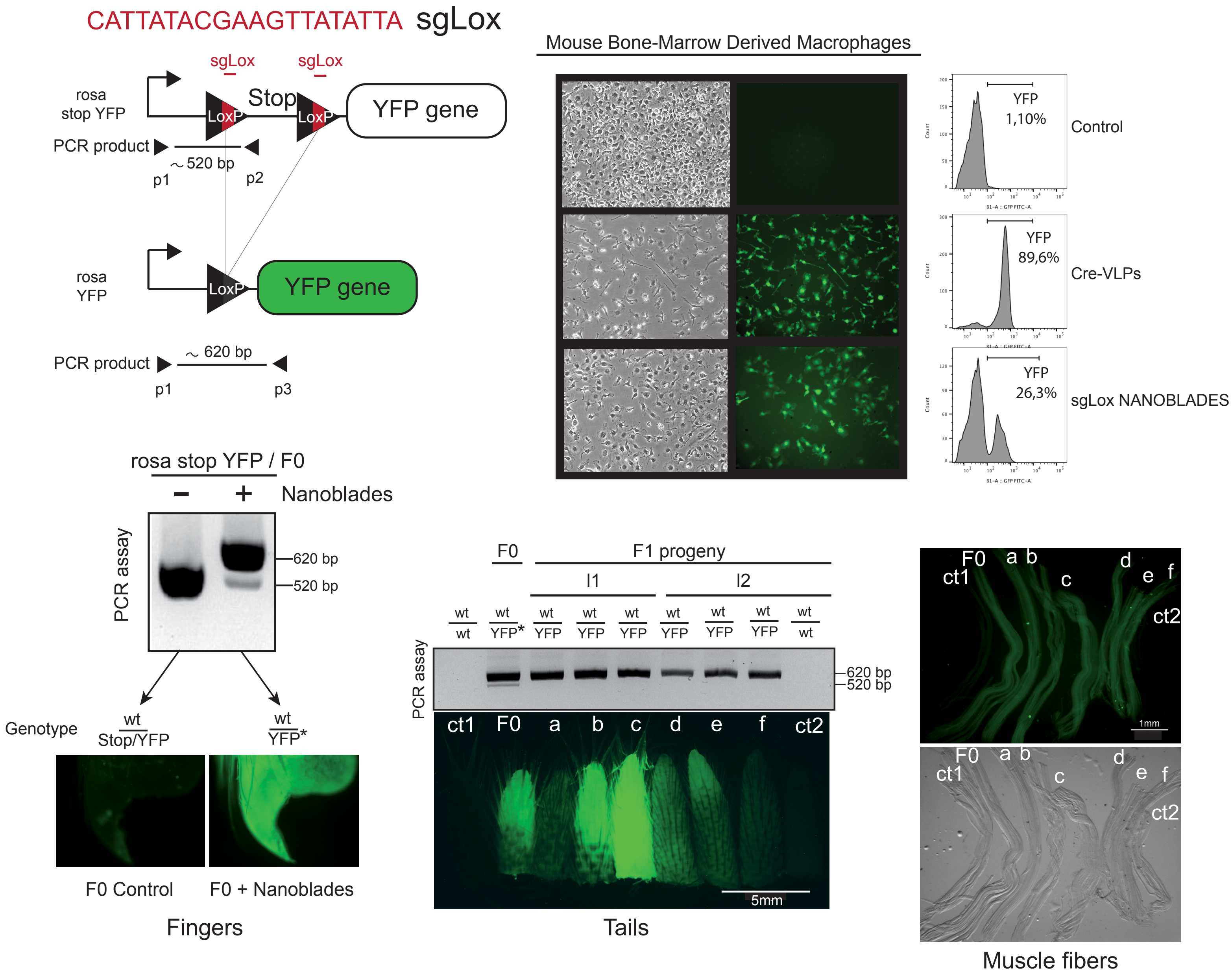

Supplementary figure 5. Generation of transgenic mice using Nanoblades. Top left panel, scheme describing the inducible YFP lox cassette in the ROSA locus of C57BL/6J. Top right panel, Fluorescence microscopy and FACS analysis of BMDMs derived from mouse bone marrow cells transduced with Nanoblades programmed with anti-LoxP sgRNAs. Bottom left panel, genetic (PCR) and phenotypic (fluorescence microscopy) analysis of an F0 mouse obtained after injection of ROSA stop YFP mouse oocytes with Nanoblades programmed with anti-LoxP sgRNAs. Bottom middle and right panel, Genetic (PCR) and phenotypic (fluorescence miscroscopy) analysis of the tail and muscle fibers from F1 mice derived from the Nanoblade-treated F0 rosa stop YFP individual.

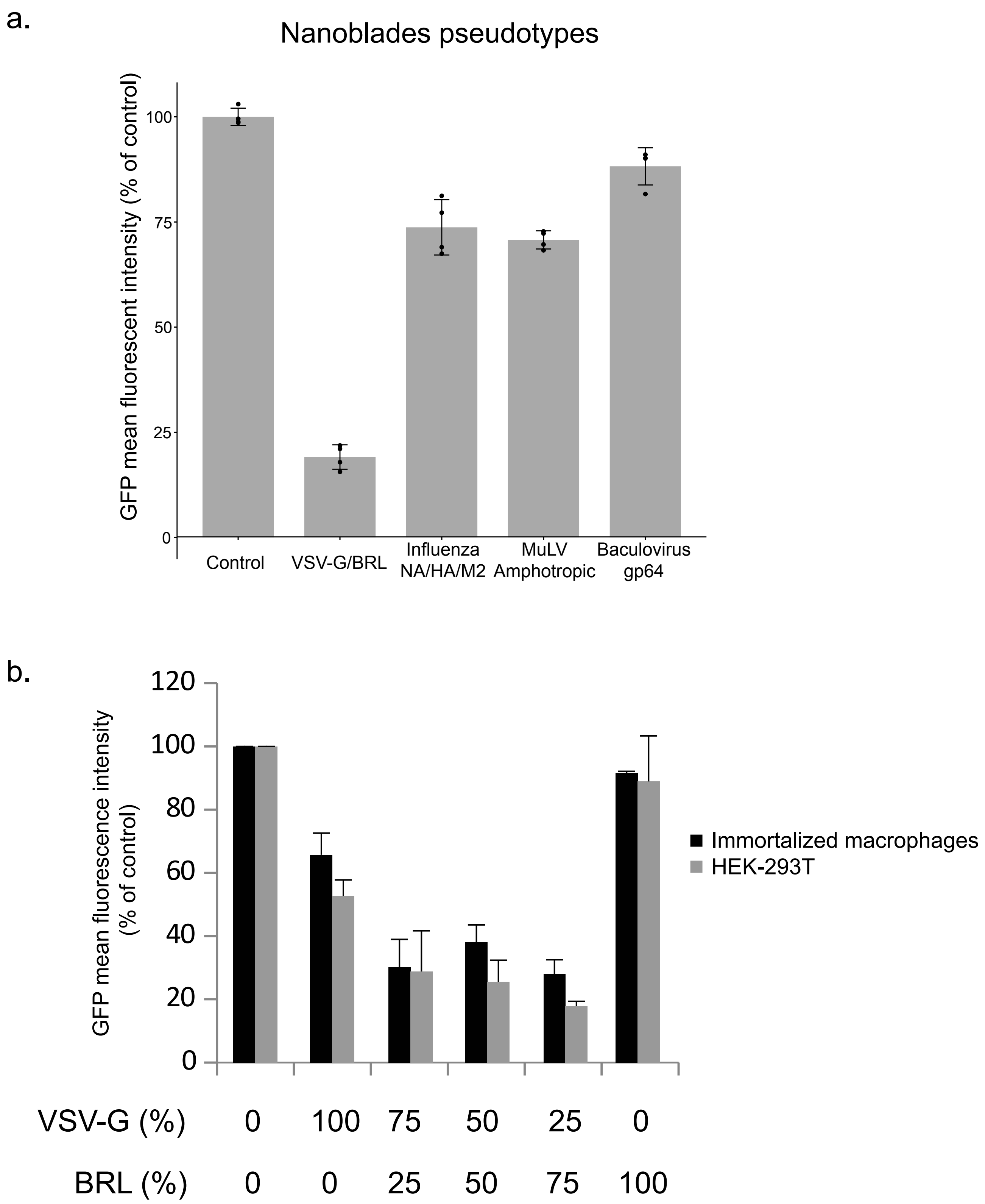

Supplementary figure 6. Pseudotyping of Nanoblades with different envelope glycoproteins. a. Nanoblades programmed with a GFP targeting sgRNA and pseudotyped with different viral-derived envelope glycoproteins were incubated with immortalized mouse macrophages that constitutively express GFP. 72 hours post-transduction, the mean fluorescence intensity (MFI) was measured by FACS (n=3, error bars correspond to standard deviation). **b.** Nanoblades programmed with a GFP targeting sgRNA and pseudotyped with different ratios of the VSV-G and BRL envelope glycoproteins were incubated with immortalized mouse macrophages and HEK293T cells that constitutively express GFP. 72 hours post-transduction, the mean fluorescence intensity (MFI) was measured by FACS (n=3, error bars correspond to standard deviation).

Supplementary Figure 7

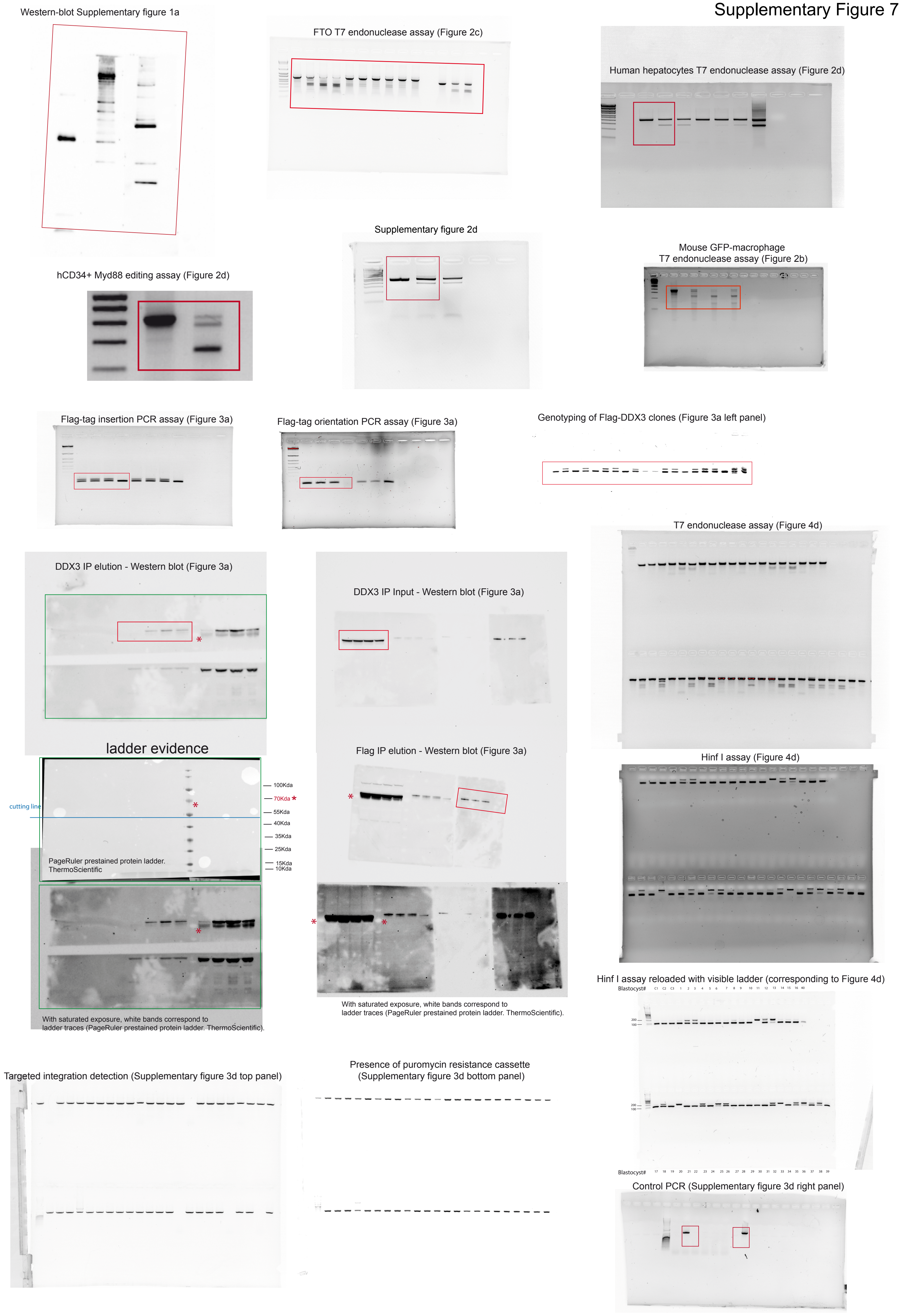

Supplement: Supplementary file 1 — Supplementary Information [file 41467_2018_7845_MOESM1_ESM.pdf]
