# Supplementary material for: Genome editing in primary cells and in vivo using viral-derived Nanoblades loaded with Cas9-sgRNA ribonucleoproteins
Source: Nat Commun. 2019 Jan 3;10:45. doi: 10.1038/s41467-018-07845-z (PMC6318322; doi:10.1038/s41467-018-07845-z)
Supplement: Supplementary file 2 — Supplementary Data 2 [file 41467_2018_7845_MOESM2_ESM.docx]

**Oligomers and sequences used in this study**

**Sequence of sgRNAs:**

Human *AAVS1:* 5’ ACCCCACAGTGGGGCCACTAggg 3’

Human *DDX3*: 5’ AGGGATGAGTCATGTGGCAGtgg 3’

Human *EMX1*: 5’ GAGTCCGAGCAGAAGAAGAAggg 3’

Human *MYD88* #1 : 5’ GAGACCTCAAGGGTAGAGGTggg 3’

Human *MYD88* #2 : 5’ GCAGCCATGGCGGGCGGTCCtgg 3’

Human rDNA: 5’ CCTTCTCTAGCGATCTGAGagg 3’

Human *TTN* -169: 5’ CCTTGGTGAAGTCTCCTTTGagg 3’

Human *TTN* -252: 5’ ATGTTAAAATCCGAAAATGCagg 3’

Human *TTN* -326: 5’ GGGCACAGTCCTCAGGTTTGggg 3’

Human *TTN* -480: 5’ ATGAGCTCTCTTCAACGTTAagg 3’

Mouse *Fto:* 5’ CATGAAGCGCGTCCAGACCGcgg 3’

Mouse *Hpd*: 5’ GAGTTTCTATAGGTGGTGCTGGGTGggg 3’

Mouse *Tyr*: 5’ GGGTGGATGACCGTGAGTCCtgg 3’ Obtained from Chen et al., 2016

Mouse Tyr: 5’ AACTTCATGGGTTTCAACTGcgg 3’ Obtained from Yoon et al., 2018

Mouse *Tyr:* 5’ ATGGGTGATGGGAGTCCCTGcgg 3’ This study.

*LoxP*: 5’ CATTATACGAAGTTATATTAagg 3’

*GFP*: 5’ CGAGGAGCTGTTCACCGGGGtgg 3’

**Sequence of oligomers used for in vitro transcription of sgRNAs:**

Human *EMX1*:

5’ TTCTAATACGACTCACTATAgagtccgagcagaagaagaaGTTTTAGAGCTAGA 3’

Mouse *Fto:*

5’ TTCTAATACGACTCACTATAgcatgaagcgcgtccagaccgGTTTTAGAGCTAGA 3’

**Sequence of primers used for homology-directed recombination experiments:**

Flag-DDX3 primer:

5’-ACTCGCTTAGCAGCGGAAGACTCCGagTTCTCGGTACTCTTCAGGGATGGA

CTACAAGGACGACGATGACAAGagTCATGTGGCAGTGGAAAATGCGCTCGGGCTGGACCAGCAGGTGA-3’

**Primers used for PCR amplification:**

Human-*DDX3*-Forward: 5'-CTTCGCGGTGGAACAAACAC-3'

Human-*DDX3*-Reverse1 5'-CGCCATTAGCCAGGTTAGGT-3'

Flag-Forward: 5’-GACTACAAGGACGACGATGACAAG-3’

Human-*DDX3*-Reverse2: 5'-CGCCATTAGCCAGGTTAGGT-3'

GFP-Forward (for T7 endonuclease assay): 5’-TAGAGCCTCTGCTAACCATGTTCATG-3’

GFP-Reverse (for T7 endonuclease assay): 5’-GAAGAAGTCGTGCTGCTTCATGTGGTC-3’

Human-*MYD88* Forward: 5’-AGATTCCTACTTCTTACGCCCCCC-3’

Human-*MYD88* Reverse: 5’-GTCTCCAGTTGCCGGATCTCCAAG-3’

Puromycin-Forward 1: 5’-GGCAGGTCCTGCTTTCTCTGAC-3’

Puromycin-Forward 2: 5’-GATATACGCGTCCCAGGGCCGGTTAATGTGGCTC-3’

Puromycin-Reverse 1: 5’-GATCCAGATCTGGTGTGGCGCGTGGCGGGGTAG-3’

Human-AAVS1-Forward:5’-CGGAACTCTGCCCTCTAACGCTG-3’

Puromycin reverse 2:5’-GATCCAGATCTGGTGTGGCGCGTGGCGGGGTAG-3’

Puromycin reverse 3: 5’-CACCGTGGGCTTGTACTCGGTCAT-3’

Mouse-*Fto*-Forward: 5’-GTAAACTGAGGCTCGAGAGTGGCTAC-3’

Mouse-*Fto*-Reverse: 5’-CACCTCACAGAGTACTCACCTCC-3’

Mouse-*Hpd*-Forward 1: 5'-CTTAGGAGGTTAGCCAAAGATGGGAG-3'

Mouse-*Hpd*-Reverse 1: 5'-TCTAGTCTCTATCCAGGGCTCCAGCC-3'

Mouse-*Hpd*-Forward 2: 5'-GAACTGGGATTGGCTAGTGCG-3'

Mouse-*Hpd-*Reverse 2: 5'-CACCCAGCACCACCTATAGAAACTC-3'

Mouse-*Tyr*-Forward 1 (for T7 endonuclease assay): 5’-TCTTTTCGGAGACACTCAAATCA-3’ Obtained from Chen et al., 2016

Mouse-*Tyr*-Reverse 1 (for T7 endonuclease assay): 5’-GCTTTCAGGCAGAGGTTCCT-3’ Obtained from Chen et al., 2016

Mouse-*Tyr*-Forward 2 (for T7 endonuclease assay): 5’-TCTGTACAATTTGGGCCCCC-3’ Obtained from Chen et al., 2016

Mouse-*Tyr*-Forward 3 (for mouse genotyping): 5’-GTGACACTCATTAACCTATTGGTGCAG-3’

Mouse-*Tyr*-Reverse 2 (for mouse genotyping): 5’-GTGACACTCATTAACCTATTGGTGCAG-3’

ROSA26-*loxP*-UPS (detection of LoxP cassette): 5’-AAGGGAGCTGCAGTGGAGTA-3’

ROSA26-*loxP*-PGK (detection of LoxP cassette): 5’-GCCAGAGGCCACTTGTGTAG-3’

ROSA26-loxP-YFP (detection of LoxP cassette): 5’-TGGTGCAGATGAACTTCAGG-3’

Human-*EMX1*-Forward (for T7 endonuclease assays): 5’-TTCTCTCTGGCCCACTGTGTCCTC-3’

Human-*EMX1*-Reverse (for T7 endonuclease assays): 5’-AGCCCATTGCTTGTCCCTCTGTCAATG-3’

Human-EMX1-Forward (for high-throughput sequencing of the locus):

5’-ACACTCTTTCCCTACACGACGCTCTTCCGATCTGGTTCCAGAACCGGAGGACAAAGTAC-3’

Human-*EMX1*-Reverse (for high-throughput sequencing of the locus):

5’-GTGACTGGAGTCCTCTCTATGGGCAGTCGGTGAAGCCCATTGCTTGTCCCTCTGTCAATG-3’

Human-*MFAP1-*Forward (for high-throughput sequencing of the locus):

5’-ACACTCTTTCCCTACACGACGCTCTTCCGATCTCCATCACGGCCTTTGCAAATAGAGCCC-3’

Human-MFAP1-Reverse (for high-throughput sequencing of the locus):

5’-GTGACTGGAGTCCTCTCTATGGGCAGTCGGTGACAGAGGGAACTACAAGAATGCCTGAGC-3’

Miseq-Custom 1: 5’ ATCACCGACTGCCCATAGAGAGGACTCCAGTCAC 3’

Miseq-Custom 2: 5’ GTGACTGGAGTCCTCTCTATGGGCAGTCGGTGAT 3’

**Primers used for quantitative PCR assays:**

Human-*DNMT3B* -Forward: 5’-ATAAGTCGAAGGTGCGTCGT-3’

Human-*DNMT3B* -Reverse: 5’-GGCAACATCTGAAGCCATTT-3’

Human-*OCT4*-Forward: 5’-CCTCACTTCACTGCACTGTA-3’

Human-*OCT4*-Reverse: 5’-CAGGTTTTCTTTCCCTAGCT-3’

Human-*NANOG*-Forward: 5’-CAAAGGCAAACAACCCACTT-3’

Human-*NANOG*-Reverse: 5’-TCTGCTGGAGGCTGAGGTAT-3’

Human-*SOX2*-Forward: 5’-CCGGTACGCTCAAAAAGAAA-3’

Human-*SOX2*-Reverse: 5’-TGTCATTTGCTGTGGGTGAT-3’

Human-TTN-Forward: 5’-TGTTGCCACTGGTGCTAAAG-3’

Human-TTN-Reverse: 5’-ACAGCAGTCTTCTCCGCTTC-3’

Human-GAPDH-Forward: 5’-ACCACAGTCCATGCCATCACT-3’

Human-GAPDH-Reverse: 5’-CCATCACGCCACAGTTTCC-3’

Mouse-*Cxcl10*-Forward: 5’-GCTGCCGTCATTTTCTGC-3’

Mouse-*Cxcl10*-Reverse: 5’-TCTCACTGGCCCGTCATC-3’

Mouse-*Cxcl2*-Forward: 5’-CCCTGGTTCAGAAAATCATCC-3’

Mouse-*Cxcl2*-Reverse: 5’-CTTCCGTTGAGGGACAGC-3’

Mouse-*Il6*-Forward: 5’-GCTACCAAACTGGATATAATCAGGA-3’

Mouse-*Il6*-Reverse: 5’-CCAGGTAGCTATGGTACTCCAGAA-3’

Mouse-*Tnf*-Forward: 5’-CTGTAGCCCACGTCGTAGC-3’

Mouse-*Tnf*-Reverse: 5’-TTGAGATCCATGCCGTTG-3’

Mouse-*Hprt*-Forward: 5’-TCATTATGCCGAGGATTTGGA-3’

Mouse-*Hprt*-Reverse: 5’-CAGAGGGCCACAATGTGATG-3’

**Oligomer used as a probe for northern-blotting of sgRNAs:**

sgRNA antisense probe:

5’GCACCGACTCGGTGCCACTTTTTCAAGTTGATAACGGACTAGCCTTATTTTAACTTGCTATTTCTAGCTCTA3’
