# Supplementary material for: Genome editing in primary cells and in vivo using viral-derived Nanoblades loaded with Cas9-sgRNA ribonucleoproteins
Source: Nat Commun. 2019 Jan 3;10:45. doi: 10.1038/s41467-018-07845-z (PMC6318322; doi:10.1038/s41467-018-07845-z)
Supplement: Supplementary file 4 — Description of Additional Supplementary Files [file 41467_2018_7845_MOESM4_ESM.docx]

**Title:** Supplementary data file 1.
**Description:** Mass spectrometry analysis of purified Nanoblades.

**Title:** Supplementary data file 2.
**Description:** Primers, oligomers and sequences used in this study
