# Supplementary material for: Genome editing in primary cells and in vivo using viral-derived Nanoblades loaded with Cas9-sgRNA ribonucleoproteins
Source: Nat Commun. 2019 Jan 3;10:45. doi: 10.1038/s41467-018-07845-z (PMC6318322; doi:10.1038/s41467-018-07845-z)
Supplement: Supplementary file 6 — Reporting Summary [file 41467_2018_7845_MOESM6_ESM.pdf]

nature research

Corresponding author(s): Emiliano Ricci

Reporting Summary

Nature Research wishes to improve the reproducibility of the work that we publish. This form provides structure for consistency and transparency in reporting. For further information on Nature Research policies, see Authors & Referees and the Editorial Policy Checklist.

Statistical parameters

When statistical analyses are reported, confirm that the following items are present in the relevant location (e.g. figure legend, table legend, main text, or Methods section).

n/a	Confirmed	
☐	☒	The exact sample size (*n*) for each experimental group/condition, given as a discrete number and unit of measurement
☐	☒	An indication of whether measurements were taken from distinct samples or whether the same sample was measured repeatedly
☐	☒	The statistical test(s) used AND whether they are one- or two-sided<br>*Only common tests should be described solely by name; describe more complex techniques in the Methods section.*
☒	☐	A description of all covariates tested
☒	☐	A description of any assumptions or corrections, such as tests of normality and adjustment for multiple comparisons
☐	☒	A full description of the statistics including central tendency (e.g. means) or other basic estimates (e.g. regression coefficient) AND variation (e.g. standard deviation) or associated estimates of uncertainty (e.g. confidence intervals)
☒	☐	For null hypothesis testing, the test statistic (e.g. *F*, *t*, *r*) with confidence intervals, effect sizes, degrees of freedom and *P* value noted<br>*Give P values as exact values whenever suitable.*
☒	☐	For Bayesian analysis, information on the choice of priors and Markov chain Monte Carlo settings
☒	☐	For hierarchical and complex designs, identification of the appropriate level for tests and full reporting of outcomes
☒	☐	Estimates of effect sizes (e.g. Cohen's *d*, Pearson's *r*), indicating how they were calculated
☐	☒	Clearly defined error bars<br>*State explicitly what error bars represent (e.g. SD, SE, CI)*

Our web collection on statistics for biologists may be useful.

Software and code

Policy information about availability of computer code

Data collection	*Provide a description of all commercial, open source and custom code used to collect the data in this study, specifying the version used OR state that no software was used.*
Data analysis	*Provide a description of all commercial, open source and custom code used to analyse the data in this study, specifying the version used OR state that no software was used.*

For manuscripts utilizing custom algorithms or software that are central to the research but not yet described in published literature, software must be made available to editors/reviewers upon request. We strongly encourage code deposition in a community repository (e.g. GitHub). See the Nature Research guidelines for submitting code & software for further information.

Data

Policy information about availability of data

All manuscripts must include a data availability statement. This statement should provide the following information, where applicable:

- Accession codes, unique identifiers, or web links for publicly available datasets
- A list of figures that have associated raw data
- A description of any restrictions on data availability

Gene Expression Omnibus: GSE107035 [https://www.ncbi.nlm.nih.gov/geo/query/acc.cgi?acc=GSE107035]
The source data underlying Figs 2b-d, 3a, 4d and Supplementary Figs 1a, 2d and 3d are provided as a Source Data file.

Field-specific reporting

Please select the best fit for your research. If you are not sure, read the appropriate sections before making your selection.

☒ Life sciences ☐ Behavioural & social sciences ☐ Ecological, evolutionary & environmental sciences

For a reference copy of the document with all sections, see nature.com/authors/policies/ReportingSummary-flat.pdf

Life sciences study design

All studies must disclose on these points even when the disclosure is negative.

Sample size	No sample size calculation was performed. Most of our experiments involved testing whether Nanoblades mediate genome editing in cultured cells where sample size is not relevant. Experiments involving animals did not require statistical analysis of the results as we were mainly testing for the ability of Nanoblades to mediate genome editing. We therefore did not perform any sample size calculation.
Data exclusions	We did not exclude any data in any of the experiments performed in cultured cells.<br>Regarding the experiments consisting in retro-orbital injection of Nanoblades in FRG mice (Figure 5), we decided to exclude results from 2 controls of the second biological replicate (Figure 5b bottom panel) to improve the clarity of the figure. Indeed, all negative controls were negative as expected, so we decided to display only 2 out of the 4 controls performed. The data is available upon request.
Replication	Most experiments performed in cultured cells were performed in three independent biological replicates.<br>Experiments involving the generation of transgenic animals (Figure 4 and Supplementary Figure 5) were performed in two independent injections of individuals. As we were looking for a binary response (gene inactivation or not) and did not plan on doing any statistical analysis, the number of injected embryos was defined in each replicate by the number of embryos available.<br>Experiments involving retro-orbital injection of Nanoblades in FRG mice (Figure 5) were performed in 2 independent biological replicates, the first consisting in the injection of 8 mice (3 controls and 5 aiming at disrupting the Hpd gene) for the first replicate and 14 mice for the second biological replicate (6 controls and 8 aiming at disrupting the HPD gene). Again, we were looking for a binary response (gene editing or not) so the number of animals used in each experiment was mainly defined by their availability.<br>Dose dependent editing of HEK293 presented in Figure 1c corresponds to a single replicate where the Cas9 amount within Nanoblades was dosed by Elisa, but the experiment was performed in additional replicates to show a dose response in genome editing.<br>Control experiments to monitor the co-delivery of cellular proteins by Nanoblades (presented in Supplementary Figure 2c and d) were performed in two independent replicates.
Randomization	Our experiments did not involve testing on a large number of individuals or groups of individuals. As such randomization of samples is not relevant to our study.
Blinding	For all animal experiments, the persons extracting total DNA and monitoring genome-editing did not have access to group allocation until we obtained the results from our tests (they only had access to the animal identification number).

Reporting for specific materials, systems and methods

Materials & experimental systems

n/a	Involved in the study
☐	☒ Unique biological materials
☐	☒ Antibodies
☐	☒ Eukaryotic cell lines
☒	☐ Palaeontology
☐	☒ Animals and other organisms
☒	☐ Human research participants

Methods

n/a	Involved in the study
☒	☐ ChIP-seq
☒	☐ Flow cytometry
☒	☐ MRI-based neuroimaging

Unique biological materials

Policy information about availability of materials

Obtaining unique materials

All plasmids required to produce Nanoblades are readily available upon request to the authors and will be also available through addgene. The plasmid coding for the genetically engineered Baboon R-less envelope that is important to produce efficient Nanoblades is available upon request from Els Verhoeyen (els.verhoyen@ens-lyon.fr).
Nanoblades are protected by a patent (WO2017068077 A1) and therefore all commercial applications derived from our materials must be licensed.

Antibodies

Antibodies used

Name, Vendor, Catalog and lot number, Validation
Anti-Gag MLV (Rat), ATCC, R187 (ATCC® CRL-1912™), validation for western-blotting: Chesebro B, et al. Characterization of monoclonal antibodies reactive with murine leukemia viruses: Use in analysis of strains of Friend MCF and Friend ecotropic murine leukemia virus. Virology 127: 134-148, 1983. PubMed: 6305011
Anti-Cas9 [7A9-3A3] (Mouse), Cell Signaling Technology, mAb #14697 - Lot number: 1, validation for western-blotting: https://www.cellsignal.com/products/primary-antibodies/cas9-7a9-3a3-mouse-mab/14697
VSV-G [P5D4] (Mouse), abcam, ab50549, validation for western-blotting: http://www.abcam.com/vsv-g-tag-antibody-p5d4-ab50549.html
Anti-Histone H2A.X (phospho S139) [EP854(2)Y] (Rabbit), abcam, ab81299, validation for immunofluorescence: http://www.abcam.com/histone-h2ax-phospho-s139-antibody-ep8542y-chip-grade-ab81299.html
Anti-RPA194 (C-1) (Mouse), Santa-Cruz Biotechnology, sc-48385, validation for immunofluorescence: https://www.scbt.com/scbt/product/rpa194-antibody-c-1
Anti-FLAG M2 (Mouse), Sigma, F1804, validation for western-blotting: Histone H1 Limits DNA Methylation in Neurospora crassa Michael Seymour, et.al G3 (Bethesda, Md.) , -, (2016)

Validation

Name, Vendor, Catalog and lot number, Validation
Anti-Gag MLV (Rat), ATCC, R187 (ATCC® CRL-1912™), validation for western-blotting: Chesebro B, et al. Characterization of monoclonal antibodies reactive with murine leukemia viruses: Use in analysis of strains of Friend MCF and Friend ecotropic murine leukemia virus. Virology 127: 134-148, 1983. PubMed: 6305011
Anti-Cas9 [7A9-3A3] (Mouse), Cell Signaling Technology, mAb #14697 - Lot number: 1, validation for western-blotting: https://www.cellsignal.com/products/primary-antibodies/cas9-7a9-3a3-mouse-mab/14697
VSV-G [P5D4] (Mouse), abcam, ab50549, validation for western-blotting: http://www.abcam.com/vsv-g-tag-antibody-p5d4-ab50549.html
Anti-Histone H2A.X (phospho S139) [EP854(2)Y] (Rabbit), abcam, ab81299, validation for immunofluorescence: http://www.abcam.com/histone-h2ax-phospho-s139-antibody-ep8542y-chip-grade-ab81299.html
Anti-RPA194 (C-1) (Mouse), Santa-Cruz Biotechnology, sc-48385, validation for immunofluorescence: https://www.scbt.com/scbt/product/rpa194-antibody-c-1
Anti-FLAG M2 (Mouse), Sigma, F1804, validation for western-blotting: Histone H1 Limits DNA Methylation in Neurospora crassa Michael Seymour, et.al G3 (Bethesda, Md.) , -, (2016)

Eukaryotic cell lines

Policy information about cell lines

Cell line source(s)

The HEK293-LentiX cell-line used for Nanoblades production and transduction tests was purchased from Clontech (Catalog number:632180) specifically for this project.
Primary Human fibroblasts were purchased from Coriell Institute for Medical Research (Catalog number: GM00321).
Primary human hepatocytes were purchased from BD Biosciences (Catalog number 454541, lot number: 342).
U2OS cells were obtained from a collaborator.

Authentication

Most of our cell lines were bought from commercial vendors specifically for this project. We therefore did not perform any authentication test. U2OS cells used in Figure 1 have not been authenticated.

Mycoplasma contamination

All immortalized cell lines used in this study were tested negative for mycoplasma contamination using a commercial kit (MycoAlert™ Mycoplasma Detection Kit, catalog number: LT07).

Commonly misidentified lines (See ICLAC register)	None of the cell lines used in our study are listed in the ICLAC database of commonly misidentified cell lines.

Animals and other organisms

Policy information about studies involving animals; ARRIVE guidelines recommended for reporting animal research

Laboratory animals	Mouse, NOD FRG mice (Fah-/-/Rag2-/-/Il2rg-/-) (Yecuris corporation), female, 12 weeks old.<br>Mouse, FVB/NRj (Janvier Biolabs), female, 4 or 5 week old.<br><br>Mouse, B6D2F1, male, adult.
Wild animals	The study did not involve the use of wild animals.
Field-collected samples	The study did not involve samples collected from the field.
